# The Bowel Microbiota and Inflammatory Bowel Diseases

**DOI:** 10.4061/2010/954051

**Published:** 2010-08-05

**Authors:** Gerald W. Tannock

**Affiliations:** Department of Microbiology and Immunology, University of Otago, P.O. Box 56, Dunedin 9054, New Zealand

## Abstract

The human bowel contains a large and biodiverse bacterial community known as the microbiota or microbiome. It seems likely that the microbiota, fractions of the microbiota, or specific species comprising the microbiota provide the antigenic fuel that drives the chronic immune inflammation of the bowel mucosa that is characteristic of Crohn's disease and ulcerative colitis. At least twenty years of microbiological research have been expended on analysis of the composition of the bowel microbiota of inflammatory bowel disease patients in comparison to that of control subjects. Despite extensive speculations about the aetiological role of dysbiosis in inflammatory bowel diseases, knowledge that can be easily translated into effective remedies for patients has not eventuated. The causes of this failure may be due to poorly defined and executed bacteriological studies, as well as the overwhelming complexity of a biome that contains hundreds of bacterial species and trillions of bacterial cells.

## 1. Introduction

The large bowels of mammals comprise a biome (ecosystem) that includes a bacterial community that is biodiverse and numerous. It can be estimated that there are a total of twenty trillion bacterial cells per human colon if it is assumed that the colonic contents weigh on average about 200 grams. The majority of these bacteria are obligate anaerobes. They are commonly referred to as commensals or symbionts because they form long-lasting, interactive associations with their animal hosts [1, 2]. These associations are generally regarded to involve mutually beneficial interactions, although some commensals are opportunistic pathogens when appropriate predisposing events occur [3]. The bacterial communities (microbiota, microbiome) of human bowels contain predominantly representatives of four phyla: *Actinobacteria*, *Proteobacteria*, *Bacteroidetes*, and *Firmicutes *(Table 1). The *Firmicutes* and *Bacteroidetes* are numerically dominant in the community. Numerous genera and species of bacteria are represented in each of these broad phylogenetic groups and, at least in the case of humans, there is person-to-person variation in the composition of the bowel microbiota [4–6]. Descriptions of a “normal range” of bowel inhabitants of humans in terms of genera and species has recently begun to emerge, sometimes referred to as the “core” microbiota (5, 6; Table 2). The bowel microbiota is, like bacterial communities in general, self-regulating and this provides temporal stability in composition. At least in health, the proportions of broad phylogenetic groups of bacteria within the community remain much the same over time [4]. Inducible biochemical pathways that mediate hydrolysis of exogenous (dietary plant cell wall polysaccharides) and endogenous (mucins) substrates enable the bacteria to obtain an uninterrupted supply of carbon and energy despite daily changes in the composition of the human diet [7, 8]. The fermentation of hydrolytic products by the bacteria produces short chain fatty acids, amines, phenols, indoles, and gases [9]. Knowledge of the nutritional niches of specific bacterial groups is sparse but a detailed description and importance of the butyrate-producing species in the ecology of the bowel is emerging due to work at the Rowett Institute, Scotland [10, 11]. Partitioning of bacteria within the contents of the bowel occurs. Some bacterial types are more likely to be associated with particulate (plant residues) material in the digesta than in the liquid fraction [12]. The mucus associated with the mucosal surface has a layered conformation and minimizes bacterial contact with the surface of enterocytes. The outer mucus layer is loosely structured and contains bacterial cells that are perhaps living there or are trapped cells that will eventually be swept away by the mucus flow. The inner mucus layer is dense and devoid of bacteria in healthy people [13]. Nevertheless, bacteria-enterocyte signaling has been demonstrated in laboratory animal models [14]. This suggests that at least bacterial cell components or products pass through the mucus and reach receptors on the surface of enterocytes. These signaling events have been demonstrated to affect physiological processes even in systemic organs [15], as well as affecting mucosal immunology in which enterocytes and dendritic cells act as front-line mediators [16, 17]. Thus, the complex, numerous, and interactive microbiota of the bowel cannot be overlooked in considerations of the aetiology of inflammatory bowel diseases. 

Crohn's disease (CD) and ulcerative colitis (UC) are chronic immune inflammatory conditions of the alimentary tract referred to collectively as inflammatory bowel diseases (IBD). There are strong genetic associations with susceptibility to CD in particular [18]. Environmental factors such as stress and diet are probably also important in the aetiology of IBD, but remain poorly delineated [19]. CD lesions can occur in upper regions of the alimentary tract but are usually located where there are microbial residents (ileum and colon), whereas UC is limited to the large bowel. In both diseases, therefore, a case can be made that the sheer antigenic load represented by trillions of bacterial cells will have an important role in driving chronic inflammation of the bowel mucosa [20, 21]. Experimental animal models of colitis do not exactly mimic CD or UC but can be used to examine the role of specific bacteria in the etiology of colitis in general terms. The results of this work provide the clearest evidence that bacteria resident in the bowel have an essential role in the pathogenesis of colitis because, when maintained germfree, the animals do not develop disease [22]. Two major reviews of publications relating to bacterial involvement in inflammatory bowel diseases have been published recently [23, 24]. Therefore this paper provides an appraisal of current views rather than a definitive coverage of the whole scientific literature.

## 2. Analysis of the Bowel Microbiota

Total microscope counts of the bacteria in human faces are at least two-fold greater than colony counts obtained by nonselective agar cultures [25]. Experiments using molecular exclusion probes show that the majority of the bacterial cells in feces are alive [26]. The results of nucleic acid-based analytical methods applied to bulk DNA extracted from stool or bowel samples (Table 3) have provided evidence that the discrepancy between total and colony counts is due to the presence of not-yet-cultivated bacteria [27]. In addition to providing a means of analysis of bacterial communities containing noncultivable members, culture-independent methods based on bacterial nucleic acids are useful because faecal and other samples can be collected, frozen, and dispatched to an analytical laboratory that may be far distant from the location in which the human subjects reside. Bacterial culture, on the other hand, requires that samples be processed without freezing within a few hours of collection. Thus, nucleic acid-based analyses of the bowel microbiota have held sway during the past two decades and have enabled extensive phylogenetical analysis of bowel communities to be made [6, 28]. The major weakness of nucleic acid-based analytical methods, however, is that they do not differentiate between DNA from bacteria that are actually active members of the community and transients or relatively inactive cells; DNA sequences from dead, quiescent, and metabolically active bacterial cells are all detected by methods based on the extraction of bulk DNA from human faeces [29]. Further, bacterial metagenomic sequences garnered from the bowel biome show the metabolic potential of the microbiota and its phylogenetic composition, but cannot reveal the temporal changes in expression of bacterial genes in the bowel, although this will be achievable through metatranscriptomics studies [8, 28]. While culture-independent studies have been indispensable in modern microbial ecological studies and have yielded a vast amount of information about the phylogeny and metabolic potential of the bowel microbiota, there is a need to return to culture-dependent studies. Such studies have been largely abandoned in recent times because so many of the bacterial inhabitants of the bowel were considered to be unculturable. New enrichment culture methods hold hope that the so far uncultivated bacteria will soon be cultured. Culture of bowel bacteria that have specific metabolic or antigenic properties would greatly enhance gene knockout or transgenic, gnotobiotic animal studies. Ranking bowel bacteria in terms of proinflammatory potential might be a worthwhile exercise and could probably only be achieved with cultivated species in combination with in vitro or in vivo immunological systems. The immune system of CD and UC patients has been clearly shown to be dysregulated and that this is associated with mutations in distinct genetic loci [20, 21]. Therefore, the balance between the bacterial inhabitants of the bowel and the mucosal immune system has to be considered to be different to that prevailing in healthy subjects. This assumption must underpin all considerations of the role of the bowel microbiota in inflammatory bowel diseases.

## 3. Confounding Factors in Analysis of the Microbiota

Bacteriological analysis of the bowel microbiota is fraught with several difficulties that often confound valid interpretation of results.

### 3.1. Sampling

Bacterial communities in stool reflect the bacteriology of the rectum and do not offer much ecological knowledge of other regions of the digestive tract [30]. Mucosal biopsies, unlike stool, provide samples collected from regions of the intestinal tract where inflammation occurs. They are not perfect specimens for bacteriological analysis, however, because they consist of only a few milligrams of tissue and have been collected from subjects that have usually undergone bowel cleansing prior to endoscopy. Residual bowel cleansing solution pools in the large bowel and can be collected by aspiration. The aspirate, essentially a faecal solution, bathes the mucosal surface of the intestine and in all likelihood contaminates it, as well as contaminating the endoscope and its mechanical parts that collect the tissue sample. Despite washing of the biopses immediately after collection, the TTGE profiles of biopsy-, aspirate-, and faecal bacteria have been reported to be highly (about 80% on average) similar [31]. This result supports the view that bacteria detected in association with biopsies are mostly contaminants from a faecal solution (aspirate) that pools in the bowel and bathes the mucosal surface after bowel cleansing.

### 3.2. Individuality and Nationality of Subjects

The composition of bowel communities, as judged by the results of stool and biopsy analysis, is individualistic, extending even down to the level of bacterial strains [32, 33]. Adding to the complexity of the situation, it has been shown that TTGE profiles generated from bacteria associated with biopsies were influenced by nationality of the donors: Mexican biopsy-associated profiles could be differentiated from those of Canadians [31]. This might be a particularly important observation because Canada has the highest incidence and prevalence of Crohn's disease yet reported, whereas this disease is rare in Mexico [34, 35]. Thus, the bowel community of Canadians may contain commensals critical to fueling chronic immune inflammation, whereas most Mexicans may lack them. It was noteworthy that TTGE profiles of Canadians commonly contained DNA fragments originating in members of the *Bacteroidetes*, a bacterial phylum that has been detected more commonly in association with biopsies of Crohn's disease patients, as well as producing bowel inflammation in an experimental animal model of colitis [36, 37].

### 3.3. Choice of Subjects

Most studies involving bowel commensals have been weak in terms of statistical power because only small numbers of patients are studied [24]. Carefully matched patients and controls need to be recruited, and sampling of patients and controls on several occasions would provide more reliable results. Previous or concurrent therapeutic drugs administered to patients, although not antimicrobial, may nevertheless alter bowel physiology, and hence community composition, relative to that of controls.

## 4. The Bowel Microbiota and Inflammation

There are several ways in which the microbiota might be linked to Crohn's disease or ulcerative colitis: the microbiota as a whole could act as a surrogate pathogen; specific members of the microbiota could be overt pathogens and incite mucosal inflammation; changes in the proportions of phylogenetic groups comprising the microbiota could initiate or perpetuate the inflammation by providing a pathogenic, antigenic fuel. Alternatively, changes in composition could remove members of the microbiota that normally inure the mucosal immune system to the presence of commensals in the bowel.

### 4.1. The Unaltered Microbiota Acts as a Surrogate Pathogen

This hypothesis concerning the pathogenesis of inflammatory bowel diseases does not invoke changes to the bacteriology of the bowel. It is supported by the observation that the bacterial profiles associated with biopsies are not different between inflamed and noninflamed mucosa [36, 38]. In this proposition, genetic predisposition of patients to abnormal permeability of the bowel mucosa allows entry of commensal antigens into subepithelial tissues. Cells of the immune system are activated as if infection by an invasive pathogen had occurred. Impaired regulation of the subsequent immune inflammation, again due to genetic predisposition, results in a chronic immune inflammation; the immune response is poorly regulated and the permeable epithelium allows constant movement of antigens into the tissue so as to resemble a continuing infection [39]. In this scenario, it is the antigens that pass to the subepithelial tissues that are relevant, rather than phylogenetic issues. It may be more useful to identify bacterial antigens against which the immune cells of Crohn's disease and ulcerative colitis patients react rather than to determine shifts in community composition. Knowledge of these antigens might aid diagnosis, as in the case of the CBir1 flagellin against which Crohn's disease patients with complicated conditions produce high antibody titres, or be included in assays useful in the development of anti-inflammatory drugs [40].

### 4.2. Specific Members of the Microbiota Act as Overt Pathogens and Incite Mucosal Inflammation

The concentration of enterobacteria in CD may be increased [24] and they may be phenotypically different from commensal *Escherichia coli*. Darfeuille-Michaud et al. isolated *E. coli* from resected chronic ileal lesions and from neoterminal ileum (with and without CD recurrence) after surgery [41]. Many of the isolates from diseased ileum adhered to Caco-2 cells. These authors confirmed, in a subsequent study, that an adherent-invasive type of *E. coli *(reference strain LF82) was specifically associated with ileal mucosa in some CD patients [42]. 

*Bifidobacterium animalis* has been shown to be more prevalent in colitic mice. In this study, the colonic microbiota of formerly germfree interleukin 10 (IL-10)-deficient mice that had been exposed to the faecal microbiota of specific-pathogen-free animals was screened using PCR/DGGE. The composition of the large bowel microbiota of IL-10-deficient mice changed as colitis progressed. DNA fragments originating from four bacterial populations (“*Bacteroides* sp.”, *Bifidobacterium animalis*, *Clostridium cocleatum*, enterococci) were more apparent in PCR/DGGE profiles of colitic mice relative to noncolitic animals, whereas two populations were less apparent (*Eubacterium ventriosum*, Acidophilus group lactobacilli). Specific DNA:RNA dot blot analysis showed that bifidobacterial rRNA abundance increased as colitis developed [43]. In a subsequent experiment, monoassociation of IL-10-deficient mice with *Bifidobacterium animalis* subspecies *animalis* resulted in bowel inflammation, thus fulfilling Koch's postulates [44]. In this study, a bacterial species belonging to a genus (*Bifidobacterium*) generally regarded as harmless in the bowel and possibly even “beneficial”, was shown to have pathogenic potential in immunologically dysregulated animals.

Chronic or recurrent pouchitis (CP) is the most important long-term complication leading to poor function following ileal pouch-anal anastomosis for ulcerative colitis. Antibiotic administration reduces symptoms of pouchitis indicating that bacteria have a role in pathogenesis [45, 46]. The stool microbiota of patients with pouchitis and of familial adenomatous polyposis patients has been shown to be markedly different [47]. PCR/TTGE profiles of the stool microbiota of familial adenomatous patients (*n* = 14) clustered at the 80% level of similarity, whereas those of pouchitis patients (*n* = 17) were disparate. The results of FISH analysis showed that bacteria not commonly present in human faeces, nor in the stool of familial adenomatous polyposis patients, comprised about 50% of the stool microbiota of untreated pouchitis patients. Antibiotic treatment reduced the proportion of these unknown bacteria in the stool of pouchitis patients. Therefore, chronic or recurrent pouchitis was found to be associated with a microbiota that contained bacteria not commonly associated with human faeces or FAP pouches. *Clostridium perfringens* was detected (comprising about 30% of the total bacterial community), by quantitative PCR, among these less common bacteria in symptomatic pouches of 10 out of 17 (58.8%) CP patients. This species was not detected in the same pouches when asymptomatic. *C. perfringens* was detected in some normal pouches (no inflammation), but in numbers about 30-fold lower than in pouchitis. [47; Tannock and Thompson-Fawcett, unpublished data]. Other studies have also reported the detection of *C. perfringens* in pouchitis samples from UC patients. For example, Ruseler-van Embden et al. [48]. analyzed the bacterial composition of the ileal reservoir from patients that had undergone a restorative proctocolectomy either for ulcerative colitis (*n* = 12) or familial adenomatous polyposis (*n* = 2). The study was carried out at least one year after the surgery and five patients were diagnosed with pouchitis. Two fecal samples were collected from each subject of the pouch control group (*n* = 9) with an interval of at least two months. Plate counts showed large differences in the anaerobic bacterial composition between two samples taken at different times suggesting the non-inflamed pouch had a bacterial community of unstable composition. Compared to the control group, stool from pouchitis patients contained a larger number of *Clostridium perfringens. *Gosselink et al. [49]. Monitored the fecal microbiota of patients diagnosed with ulcerative colitis and having undergone a pouch construction (*n* = 13). The aim of this study was to compare the effect of two antibiotics, metronidazole and ciprofloxacin, on the fecal microbiota at different times. The bacteriological content of the pouch was analyzed at the beginning of an inflammatory episode before antibiotic treatment, during treatment with ciprofloxacin or metronidazole, and during pouchitis-free periods. Higher numbers of *C. perfringens* and hemolytic strains of *E. coli* were observed during pouchitis episodes in 50% of the patients. Administration of metronidazole eradicated the *Clostridium perfringens*. Treatment with ciprofloxacin inhibited not only the growth of *Clostridium perfringens* but also that of coliforms, including hemolytic strains of *Escherichia coli*. Taken together, these results offer the possibility that *C. perfringens* is the aetiolgical agent of pouchitis in some patients. The absence of *C. perfringens* in non-inflamed pouches (antibiotic administration) but its presence in inflamed pouches (no antibiotic) probably satisfies Koch's third postulate (“the isolated microbe, when administered to humans or animals must cause disease”) in modified form.

Duffy et al. [50] compared the pouch bacterial content from ulcerative colitis (*n* = 10) and familial adenomatous polyposis (*n* = 7) patients. None of the patients had had a previous episode of pouch inflammation. Sulphate-reducing bacteria were exclusively detected in pouches of ulcerative colitis patients. Ohge et al. [51]) have also shown an association between sulphate-reducing bacteria and pouches. Sulphate-reducing bacteria were detected in higher numbers in active pouchitis patients (*n* = 8) in comparison to patients without a history of pouchitis (*n* = 8), patients with past episode(s) of pouchitis (*n* = 18), patients having an ongoing antibiotic treatment for pouch inflammation (*n* = 11) and familial adenomatous polyposis patients (*n* = 5). The authors observed that this particular group of bacteria was sensitive to metronidazole or ciprofloxacin treatment.

### 4.3. Changes in the Proportions of Phylogenetic Groups Comprising the Microbiota

A decrease in the number of phylotypes (reduced biodiversity) has been reported in relation to the fecal community of CD patients [52–56]. Scanlan et al. [53], for example, reported that they failed to detect members of clostridial cluster IV in 27% of CD fecal samples (PCR) but all control samples contained these bacteria (*P* < .0001). Taken together, the results of these studies showed a quantitative and a qualitative (biodiversity) reduction in representation of the *Firmicutes* phylum, and particularly clostridial cluster IV members in the feces of CD patients. This phylogenetic group contains several butyrate producing bacteria, such as *Faecalibacterium prausnitzii* [55]. Butyrate and other short chain fatty acids are believed to be important sources of energy for colonic epithelial cells and may have anti-inflammatory properties, as well as improving barrier function of the bowel epithelium [57–60]. Hence, the decrease in butyrate-producing bacteria in the colon might have an overall detrimental effect on the colonic mucosa. The major finding of metagenomic studies to date has been, therefore, the reduced biodiversity of the microbiota. It is not clear whether the reduced biodiversity initiates inflammatory bowel diseases, perpetuates the diseases, or is a result of the diseases. Nor can a change in the composition of the faecal microbiota (representing the rectum) explain CD lesions in the small bowel. A reduction in biodiversity has been reported for both CD and UC patients which suggests that the changes in microbiota composition are due to the inflamed state of the bowel. The pathology and immunology and pathogenesis of CD and UC are distinctly different and it will be surprising if the bacterial aetiology, should there be one, is the same for both diseases. Much ingenious experimentation will be required to end speculation about these changes and to produce knowledge of benefit to medical practitioners and their patients.

## 5. Conclusions

Improvements to the quality of microbiological investigations of Crohn's disease and ulcerative colitis patients will rely on the more careful selection of patients (perhaps aided by human genotyping because of the variety of genetic polymorphisms associated with Crohn's disease), the recruitment of newly diagnosed and untreated patients, and greater attention to the way in which specimens to be used in microbiological investigations are collected. Overall, more thoughtful planning of studies aimed at analysis and comparison of commensal communities are needed in order to improve microbiological studies in Crohn's disease and ulcerative colitis. Analysis of the microbiota of CD and UC patients has so far resulted in diverging views of the importance of particular bacteria in the pathogenesis of inflammatory bowel diseases. Nevertheless, strong cases for the involvement of adherent/invasive *E. coli* in ileal lesions of CD are building, and for the role of *C. perfringens* in some cases of pouchitis in ulcerative colitis patients.

Reduced biodiversity of the microbiota in CD seems, however, to be the current popular choice of aetiological significance, especially since it provides the opportunity to develop probiotic strains of *F. prausnitzii* or other butyric acid-producing bacteria. Implantation or enrichment (by concurrent prebiotic administration) of a cultivated strain in the inflamed bowel seems an unlikely proposition since the prevailing intestinal conditions associated with disease have apparently led to the observed reduction or elimination of the species. Moreover, inflamed mucosa seems to have impaired ability to utilize butyrate [61]. Culture supernatants of *F. prausnitzii* and some other butyrate-producing bacteria appear to contain anti-inflammatory substances that might be purified and used to treat patients. Detection and testing of these presumptive anti-inflammatory substances relies on the use of experimental animal models of colitis and it is not clear yet whether the research can be translated to treat human patients. Meta-analysis of seven studies that tested the effect of maintenance treatment with standard probiotics (*Lactobacillus rhamnosus* GG, *Escherichia coli* Nissle 1917, VSL#3, *Saccharomyces boulardii*) among patients with Crohn's disease in remission did not demonstrate any benefit of probiotic treatment [62]. Elahi et al. [63], conducted a meta-analysis of five trials on the effect of probiotics on pouchitis (acute, chronic, and recurrent remission). Four of these studies (conducted by the same research group in each case) utilized the multi-strain (*Lactobacillus casei*, *Lactobacillus plantarum*, *Lactobacillus acidophilus*, *Lactobacillus delbrueckii* subspecies *bulgaricus*, *Bifidobacterium longum*, *Bifidobacterium breve*, *Bifidobacterium infantis*, and *Streptococcus thermophlus*) probiotic product VSL#3. The outcome of interest in the meta-analysis was for pouchitis defined as a pouchitis disease activity index of >7.0. Pooling of the results from five trials yielded an odds ratio of 0.04 (95% confidence interval 0.01–0.14, *P* < .0001) in the probiotic group relative to the placebo group. These kinds of results continue to encourage the continuing development of probiotic therapies.

An alternative approach may be to gain information about potent antigens associated with the bowel microbiota that drive the inflammatory response. Treatment options would then be to selectively use novel or extant antibiotics to reduce the fraction of the microbiota producing the most potent antigens, or to derive drugs that would effectively block signaling pathways stimulated by these antigens. After at least twenty years interest in the microbiology of inflammatory bowel diseases without significant accrual of knowledge that can be translated to improved treatments for patients, new thinking and new research approaches are clearly indicated.

## Figures and Tables

**Table 1:** Common terms used in bowel microbial ecology.

*Phylogeny*	The history of organismal lineages as they change through time. It implies that different species arise from previous forms via descent, linking all forms of life.
*Dysbiosis*	A term generally used in relation to the bowel biome indicating an imbalance in the composition of the microbiota.
*Firmicutes*	A phylum of bacteria, most of which have gram-positive cell wall structure. The principal genera detected in human faeces are *Clostridium*, *Eubacterium*, *Anaerostipes*, *Coprococcus*, *Dorea*, *Lachnospira*, *Roseburia*, *Faecalibacterium*, *Ruminococcus*, *Subdoligranulum*, and *Coprobacillus*.
*Bacteroidetes*	A phylum of bacteria that have gram-negative cell wall structure. The principal genera detected in human faeces are *Bacteroides*, *Parabacteroides*, and *Alistipes*.
*Actinobacteria*	A phylum of gram-positive bacteria that includes, amongst others, the genera *Bifidobacterium* and *Collinsella* that are often detected as members of the bowel microbiota of humans.
*Proteobacteria*	A phylum of bacteria that includes *Escherichia coli*, a common facultatively anaerobic species in the bowel.
*Bacteroides-Prevotella* cluster	A broad phylogenetic classification comprising gram-negative, anaerobic species forming a major portion of the bowel microbiota.
Clostridial cluster XIVa.	A broad phylogenetic classification comprised of several genera and species of gram-positive bacteria, not exclusively clostridia.
Clostridial cluster IV	A broad phylogenetic classification comprised of several genera and species of gram-positive bacteria, not exclusively clostridia.
*Enrichment culture*	An understanding of the environmental conditions favored by an organism, together with genetic clues about the microbe's abilities is used to guide the design of culture media and conditions.
*Probiotic*	Live microorganisms which when administered in adequate amounts confer a health benefit on the host.
*Prebiotic*	A dietary supplement of nondigestible carbohydrate (inulin and fructo-oligosaccharides are the best known) that can be metabolized by particular bacteria in the human colon.

**Table 2:** Common species in human faeces. Seventy five bacterial species with >1% genome sequence coverage in >50% of 124 adult humans. After Qin et al. [6]

*Faecalibacterium prausnitzii *	*Bacteroides fragilis *
*Roseburia intestinalis *	*Eubacterium biforme *
*Dorea formicigenerans *	*Bacteroides eggerthii *
*Bacteroides vulgatus *	*Streptococcus thermophilus *
*Clostridium sp *	*Bacteroides capillosus *
*Bacteroides uniformis *	*Holdemania filiformis *
*Eubacterium hallii *	*Clostridium leptum *
*Bacteroides sp. *	*Prevotella copri *
*unknown sp *	*Clostridium sp.*
*Coprococcus comes *	*Bacteroides plebeius *
*Eubacterium rectale *	*Butyrivibrio crossotus *
*Ruminococcus sp. *	*Bacteroides coprocola *
*Dorea longicatena *	*Bacteroides finegoldii *
*Bacteriodes xylanisolvens *	*Clostridium bartlettii *
*Bacteroides sp. *	*Clostridium sp. *
*Bacteroides sp. *	*Escherichia coli*
*Ruminococcus torques *	*Parabacteroides johnsonii *
*Bacteroides sp.*	*Subdoligranulum variabile *
*Alistipes putredinis *	*Bacteroides intestinalis *
*Collinsella aerofaciens *	*Catenibacterium mitsuokai *
*Parabacteroides distasonis*	*Clostridium bolteae *
*Eubacterium siraeum *	*Bifidobacterium pseudocatenulatum *
*Bacteroides ovatus *	*Anaerotruncus colihominis *
*Bacteroides sp.*	*Bifidobacterium catenulatum *
*Bacteroides sp. *	*Ruminococcus gnavus *
*Bacteroides thetaiotaomicron *	*Bacteroides coprophilus *
*Bacteroides dorei *	*Bacteroides pectinophilus *
*Parabacteroides merdae *	*Gordonibacter pamelaeae gen. nov. sp. *
*Bifidobacterium longum subsp. infantis *	*Clostridium asparagiforme *
*Ruminococcus obeum *	*Clostridium nexile *
*Bifidobacterium adolescentis *	*Blautia hansenii *
*Bacteroides caccae *	*Clostridium scindens *
*Ruminococcus bromii *	*Enterococcus faecalis *
*Ruminococcus lactaris *	*Mollicutes bacterium *
*Eubacterium ventriosum *	*Bryantella formatexigens *
*Coprococcus eutactus *	*Clostridium methylpentosum *
*Akkermansia muciniphila *	
*Bacteroides stercoris *	
*Bacteroides cellulosilyticus *	

**Table 3:** Commonly used nucleic acid-based (culture-independent) analytical methods.

*PCR/denaturing electrophoretic polyacrylamide gels (PCR/DGGE, PCR/TTGE)*
DNA is extracted directly from faecal samples. Hypervariable gene sequences (most often 16S rRNA) are amplified using PCR primers that anneal with conserved sequences that span the selected hypervariable regions. One of the PCR primers has a GC-rich 5′ end (GC clamp) to prevent complete denaturation of the DNA fragments during gradient gel electrophoresis. Using 16S rRNA gene sequences as example, amplified fragments from different types of bacteria and present in the PCR product are separated using polyacrylamide gel electrophoresis. In DGGE (denaturing gradient gel electrophoresis), the double-stranded 16S fragments migrate through a polyacrylamide gel containing a gradient of urea and formamide until they are partially denatured by the chemical conditions. The fragments do not completely denature because of the GC clamp, and migration is radically slowed when partial denaturation occurs. Because of the variation in the 16S sequences of different bacterial species, chemical stability is also different; therefore, different 16S “species” can be differentiated by this electrophoretic method. Similarly, in TTGE (temporal temperature gradient electrophoresis), the 16S sequences can be separated by gradually increasing the temperature of the polyacrylamide gel during electrophoresis. Separation is achieved on the basis of differing temperature stability of the 16S fragments. These methods generate a profile of the numerically predominant members of the bacterial community. Individual fragments of DNA can be cut from DGGE/TTGE gels, further amplified and cloned, then sequenced. The sequence can be compared to those in gene databanks in order to obtain identification of the bacterium from which the 16S sequence originated. Depending on the length of the sequence, identification to at least bacterial genus can be made.

*Fluorescent in situ hybridization/fluorescence-activated cell sorting (FISH/FC)*
DNA (oligonucleotide) probes target specific rRNA sequences (16S or 23S) within ribosomes to which they hybridize. The probes are 5′ labelled with a fluorescent dye which permits both detection and quantification of specific bacterial populations. Bacterial cells within which hybridisation with a probe has occurred fluoresce and hence can be detected and counted by epifluorescence microscopy (preferably automated) or fluorescence-activated flow cytometry. Continual reassessment of the specificity and coverage of FISH probes is essential in order to update and confirm their continuing specificity and hence reliability. This is because new 16S rRNA gene sequences are constantly added to databases. Epifluoresence microscopic detection is laborious and time consuming, and manual microscopic enumeration requires careful attention by the operator. A lower detection limit of about 10^6^ bacteria per gram of faeces can be achieved. An automated method of counting fluorescent bacterial cells has been developed by coupling fluorescence microscopy to a computerized system of image analysis. Using this automated counting device, the lower detection threshold has been estimated to be 10^7^ bacteria per gram of faeces. Therefore, only the more numerous members of the bacterial community can be detected. Nevertheless, identification of individual bacterial cells, as well as morphological and topographical information are valuable characteristics of fluorescence microscopy. Combined with flow cytometry, FISH provides a high throughput quantitative and qualitative method of analysis. Flow cytometry combines quantitative and multiparametrics analysis (size, internal granularity, fluorescence signal). A lower threshold of detection of 0.4% relative to the total number of bacteria determined with the universal bacterial probe EUB338 has been demonstrated.

*Quantitative PCR*
PCR primers and fluorescent probes targeting nucleic acid sequences, usually 16S rRNA gene sequences, which are unique to particular bacterial species, are used to quantify the specific sequences in DNA extracted from faeces. Real-time quantitative PCR can be used to quantify specific populations or phylogenetic clusters using specific PCR primers and fluorescent probes. Target sequences in DNA are amplified and simultaneously quantified (as absolute number of copies, or relative amount when normalized to DNA input, or by reference to additional normalizing genes). The procedure follows the general principle of PCR but its key feature is that the amplified DNA is detected as the reaction progresses in *real time* in contrast to standard PCR where the product of the reaction is detected at its end. Two common methods for detection of products in real-time PCR are: non-specific fluorescent dyes that intercalate with any double-stranded DNA, or (2) sequence-specific DNA probes consisting of oligonucleotides that are labelled with a fluorescent reporter which permits detection only after hybridization of the probe with its complementary DNA target.

*16S rRNA gene phylogeny*
Older studies utilised PCR amplification of 16S rRNA genes from bulk DNA extracted from faeces followed by cloning the 16S rRNA gene sequences in a plasmid vector in an *Escherichia coli* host, prior to sequencing. More recently, high throughput bead/emulsion-based sequencing of PCR-amplified DNA or random sequencing of DNA fragments derived from bacterial communities in faeces (metagenome) has been used. These approaches provide catalogues of the constituent bacterial types (usually broad phylogenetic groups) of the community when analysed in relation to a databank of 16S rRNA gene sequences (such as the Ribosomal Database Project pyrosequencing pipeline tools).

*Metagenomics*
A microbial community is studied in terms of its collective genomes. Nowadays, this approach involves shotgun genome methods to sequence random fragments of DNA from microbes in a sample collected from a biome of interest. DNA is directly extracted from the sample, is broken into small fragments, and portions of these fragments are sequenced. Searches of DNA sequence databases permit collation of the sequencing information in terms of 16S rRNA genes (biodiversity), genes associated with metabolic pathways including their potential regulation, and cell structural molecules. This methodology can reveal novel and fundamental insights about the biodiversity and metabolic impacts of microbial life in biomes.

*Metatranscriptomics*
Measuring the transcriptomics (gene expression) of microbial communities in the wild. RNA (which includes mRNA) is extracted directly from samples. Ribosomal RNA, which forms the major portion of the total RNA is removed. Then, remaining RNA is converted to cDNA by reverse-transcription PCR. Random sequencing of the cDNA reveals the transcripts produced in the ecosystem. This approach has mostly been used with oceanic samples but application of the methodology to bowel samples is possible.
